# Clinical characteristics of a male child with non-classic lipoid congenital adrenal hyperplasia and literature review

**DOI:** 10.3389/fendo.2022.947762

**Published:** 2022-11-02

**Authors:** Wenli Lu, Tingting Zhang, Lidan Zhang, Xueqing Wang, Sheng Lv, Junqi Wang, Lei Ye, Yuan Xiao, Zhiya Dong, Wei Wang, Shuoyue Sun, Chuanyin Li, Ronggui Hu, Guang Ning, Xiaoyu Ma

**Affiliations:** ^1^ Department of Pediatrics, Ruijin Hospital, Shanghai Jiao Tong University School of Medicine, Shanghai, China; ^2^ Department of Pediatric Genetic and Metabolic Endocrinology, West China Second University Hospital, Sichuan University, Chengdu, Sichuan, China; ^3^ Department of Endocrine and Metabolism, Ruijin Hospital, Shanghai Jiao Tong University School of Medicine, Shanghai, China; ^4^ Cancer Center, Shanghai Tenth People’s Hospital, School of Medicine, Tongji University, Shanghai, China

**Keywords:** lipoid congenital adrenal hyperplasia, non-classic LCAH, steroidogenic acute regulatory protein, enzymatic activity, phenotype and genotype

## Abstract

**Background:**

Lipoid congenital adrenal hyperplasia (LCAH) is a rare and severe disorder that is caused by mutations in the steroidogenic acute regulatory protein (StAR). Non-classic LCAH is defined as late-onset glucocorticoid deficiency and even complete male external genitalia in 46,XY individuals. However, to date, few cases of non-classic LCAH have been reported.

**Methods:**

It was attempted to describe the clinical characteristics of a male child with complete male external genitalia in terms of age of onset, adrenal function, and biochemical indicators. Previously reported cases were also reviewed to investigate the relationship of age of onset with enzymatic activity in non-classic LCAH.

**Results:**

The patient with complete male external genitalia was diagnosed with non-classic LCAH, in which the reason for his referral to a local hospital at the of age 1.25 years was progressive skin hyperpigmentation, and plasma adrenocorticotropic hormone (ACTH) level was elevated to higher than 1,250 pg/ml. The compound heterozygous mutations c.772C>T/c.562C>T in *STAR* gene were identified *via* genetic testing. The literature review resulted in identification of 47 patients with non-classic LCAH from 36 families. The mutational analysis showed that c.562C>T mutation was prevalent in patients with non-classic LCAH, accounting for 37.2% of the total mutant alleles, which could reflect the founder effect on the non-classic LCAH population. In total, 28 46,XY patients were reported, including 22 (78.5%) cases with complete male external genitalia and six (21.5%) cases with different degrees of hypospadias.

**Conclusion:**

The clinical phenotypes of non-classic LCAH are highly variable. Routine physical examination, laboratory measurement, genetic testing, and, importantly, enzymatic activity assay may facilitate the early diagnosis of non-classic LCAH. The age of primary adrenal insufficiency (PAI) onset may not be a diagnostic basis for non-classic LCAH, and enzymatic activity assay determination may be more effective.

## Introduction

Lipoid congenital adrenal hyperplasia (LCAH) is a rare and severe disorder that is caused by mutations in the steroidogenic acute regulatory protein (StAR) (1). StAR is a cytoplasmic enzyme with 285 amino acids encoded by the *STAR* gene located on chromosome 8p11.2 and consists of seven exons. It plays an indispensable role in the delivery of cholesterol from the outer to the inner mitochondrial membrane in the initial steps of steroid synthesis (2). Patients with LCAH are typically characterized by lipoid accumulation in the adrenal glands, adrenal insufficiency in infants, and female external genitalia regardless of karyotype. Regarding several cases caused by *STAR* gene mutations, LCAH is divided into classic LCAH and non-classic LCAH according to sex reversal and age of onset (3). Non-classic LCAH is defined as late-onset glucocorticoid deficiency and even complete male external genitalia in XY individuals. The present study aimed to report the clinical and molecular characterization of a Chinese male child with complete male external genitalia who was diagnosed with non-classic LCAH. Previously reported cases were also reviewed to enhance the understanding of non-classic LCAH.

## Methods

### Patient

In the present study, the patient has undergone extensive physical and laboratory examination for the evaluation of primary adrenal sufficiency. Collection of his clinical features and laboratory data was attempted when he was initially admitted to a hospital for adrenal insufficiency. Routine examination included testing of liver and kidney functions and measurement of the levels of serum insulin-like growth factor-1 (IGF-1) and insulin-like growth factor-1-binding protein 3 (IGFBP3). Furthermore, assessment of renin-angiotensin-aldosterone system (RAAS) function and gonadal function, measurement of plasma adrenocorticotropic hormone (ACTH) and cortisol levels, and ACTH stimulation test were performed. Bone age was assessed using the Greulich–Pyle (G-P) and Tanner–Whitehouse 3 (TW3) methods. Clinical data on the first and follow-up visits were collected, including birth history, history of growth and development, physical examination, laboratory data, and radiological data.

### Molecular and enzymatic activity assays

Genomic DNA was extracted from the peripheral blood using the DNA extraction kit (Qiagen Hilden, Germany). Primers were designed according to the *STAR* gene sequence from GenBank. The *STAR* gene was amplified using polymerase chain reaction (PCR). The enzymatic activity of four mutants of the *STAR* gene was herein investigated, including some mutations that had been functionally analyzed previously or merely reported. Non-steroidogenic COS-7 cells (CRL-1651; http://www.atcc.org) were cultured in Dulbecco’s modified Eagle’s medium (DMEM; Life Technologies Corp., Carlsbad, CA, USA) supplemented with 12% fetal bovine serum and 1% antibiotics (50 units/ml penicillin and 50 μg/ml streptomycin) at 37°C in an incubator (5% CO_2_ with humidity of 95%). Cells were seeded into 12-well plates and transfected using Lipofectamine 2000 reagent (Invitrogen Inc., Carlsbad, CA, USA) with 1 μg of empty plasmid, wild-type *STAR*, or mutant *STAR* constructs together with 1 μg of F2 plasmid, which were kindly provided by Walter L. Miller, UCSF, San Francisco, USA (4), at a confluence of approximately 80%. After 48 h of transfection, the culture supernatants were collected and the pregnenolone synthesis from endogenous cholesterol or 22(R)-hydroxycholesterol was assayed with an ELISA kit (ALPCO Diagnostics Inc., Salem, NH, USA). The empty plasmid served as a negative control to measure StAR-independent steroidogenesis. In the positive control, soluble hydroxysterol 22(R)-hydroxycholesterol (5 μg/ml; Sigma-Aldrich Chemie GmbH, Munich, Germany) was added. It is widely accepted that 22(R)-hydroxycholesterol can directly enter the mitochondria for the synthesis of steroid hormones without the contribution of enzymes. Therefore, the positive control reflects the maximum production potential of various enzymes in the cells to catalyze the production of pregnenolone. All experiments were carried out in triplicate, and each experiment was repeated three times. In the positive well, cells were incubated with 3 μl 22R-hydroxycholesterol (22R-OH) as the physiological source of cholesterol pregnenolone that was synthesized from endogenous cholesterol or 22(R)-hydroxycholesterol.

### Literature review

“Congenital lipoid adrenal hyperplasia” was searched in the PubMed and Wanfang databases to retrieve English and Chinese published articles. A total of 106 articles were published until September 2021, in which the diagnosis of non-classic LCAH was confirmed by genotyping, and participants’ clinical characteristics were described.

### Statistical analysis

All data were analyzed using Microsoft Excel software.

## Results

### Case presentation

The Chinese male child was born at a gestational age of 37 weeks with complete male external genitalia (descended testes, micropenis without hypospadias). His birth weight was 3.34 kg, and his birth length was 50 cm. He had no history of adrenal insufficiency, and the only reason for his referral to a local hospital at the age of 1.25 years was progressive skin hyperpigmentation. Laboratory examination revealed plasma ACTH of more than 1,250 pg/ml (normal range, 7–65 pg/ml), cortisol 8 a.m. was 1.36 μg/dl (normal range, 6.7–22.6 μg/dl), cortisol 4 p.m. was 0.97 μg/dl (normal range, 6.7–22.6 µg/dl), and testosterone level was <0.1 ng/ml, and he was therefore diagnosed with primary adrenal insufficiency (PAI). Hydrocortisone was given as treatment for PAI. At the age of 43 months, he was referred to our department for the elevated ACTH level and a micropenis. His height and weight were 111 cm [+2.9 standard deviation (SD)] and 24 kg ( +3 SD), respectively; his blood pressure was 86/56 mmHg. Examination showed generalized hyperpigmentation of the skin. His stretched penile length was 1.5 cm, testicular volume was 3 ml, and Tanner stage of PH1 was recorded. Laboratory examination revealed that the levels of sodium and potassium were 135 (range, 130–147) and 4.22 (range, 3.5–5.1) mmol/L, and no hyperkalemia and hyponatremia were detected. Moreover, the following laboratory data were collected: plasma ACTH level of 1,744 (normal range, 12–78) pg/ml, basal 17-hydroxyprogesterone (17-OHP) level of 0.07 (normal range, 0.07–1.53) ng/ml, cortisol level of 0.2 (normal range, 6.7–22.6) μg/dl, plasma RAAS activity of 9.99 (normal range, 0.1–5.5) ng/ml/h, aldosterone (Ald) level of 0.1 (normal range, 29.4–161.5) pg/ml, angiotensin II level of 134.22 (normal range, 18–103) pg/ml, and testosterone level of 0.01 (normal range, 1.75–7.81) ng/ml. The ACTH stimulation test showed no changes in the levels of cortisol and 17-OHP (**Table 1**). Chromosome analysis revealed a 46,XY karyotype. Luteinizing hormone-releasing hormone (LHRH) stimulation test evaluated the hypothalamic-pituitary-gonadal (HPG) axis (**Table 2**), and the result indicated that the HPG axis was not activated. The ultrasound revealed that the left testis was 21 * 11 * 8 mm^3^ and the right testis was 17 * 11 * 10 mm^3^, and the adrenal ultrasound displayed normal data without enlargement.

**Table 1:** The results of ACTH stimulation test.

	0 min	30 min	60 min
Progesterone(0.14-2.06 ng/ml)	0.01	0.01	0.01
17-OHP(0.07-1.53 ng/ml)	0.07	0.03	0.04
Cortisol(6.7–22.6 μg/dl)	0.2	0.41	0.22
Dehydroepiandrosterone sulfate (5-57 μg/dl)	2.9	3.1	2.9
Androstenedione(0.15-3.10 ng/ml)	0.15	0.11	0.05
Testosterone (1.75-7.81 ng/ml)	0.01	0.01	0.01
Ald(29.4-161.5 pg/ml)	0.1	0.1	0.1

17-OHP, 17-hydroxyprogesterone; ACTH, adrenocorticotropic hormone; Ald, aldosterone.

**Table 2:** The results of LHRH stimulation test.

	0 min	30 min	60 min	90 min
Luteinizing hormone (IU/L)	0.3	3.6	2.8	1.5
Follicle-stimulating hormone (IU/L)	0.6	3.7	4.4	3.9

LHRH, luteinizing hormone-releasing hormone.

### Mutational analysis and enzymatic activity assay

**Figure 1:**
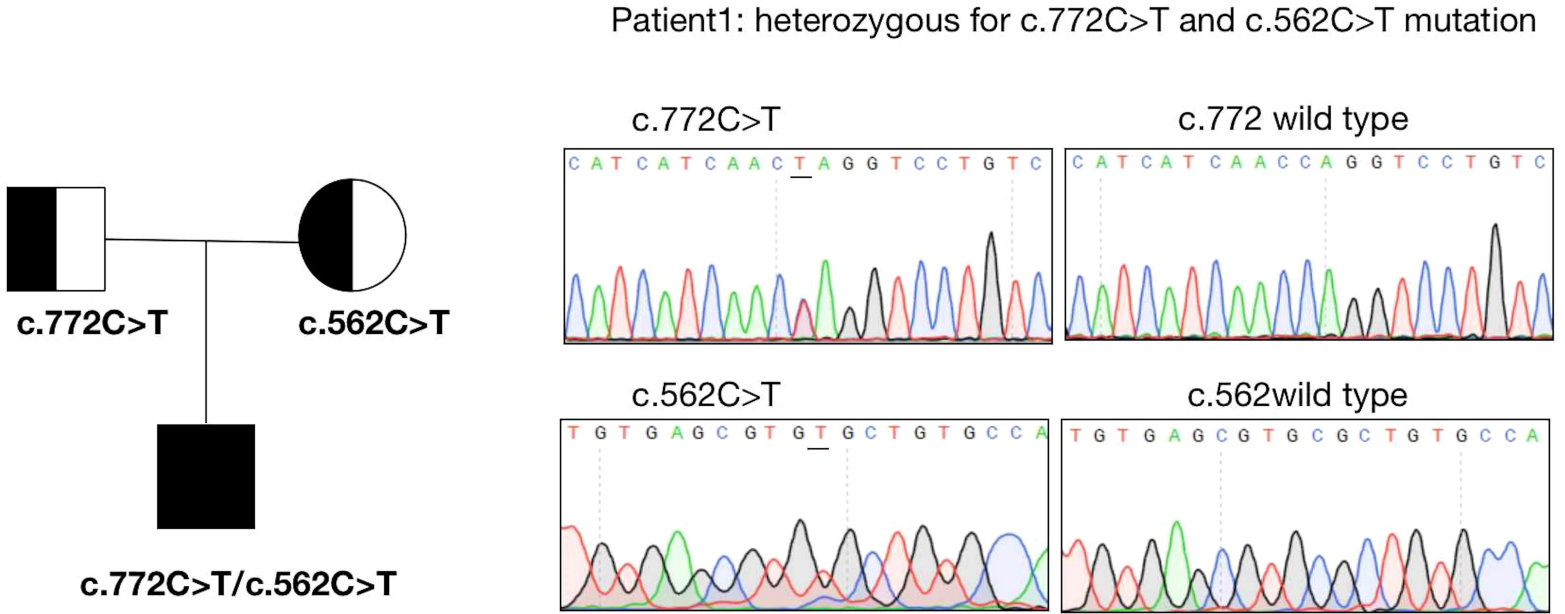
Sanger sequencing confirmation of the patient.

**Figure 2:**
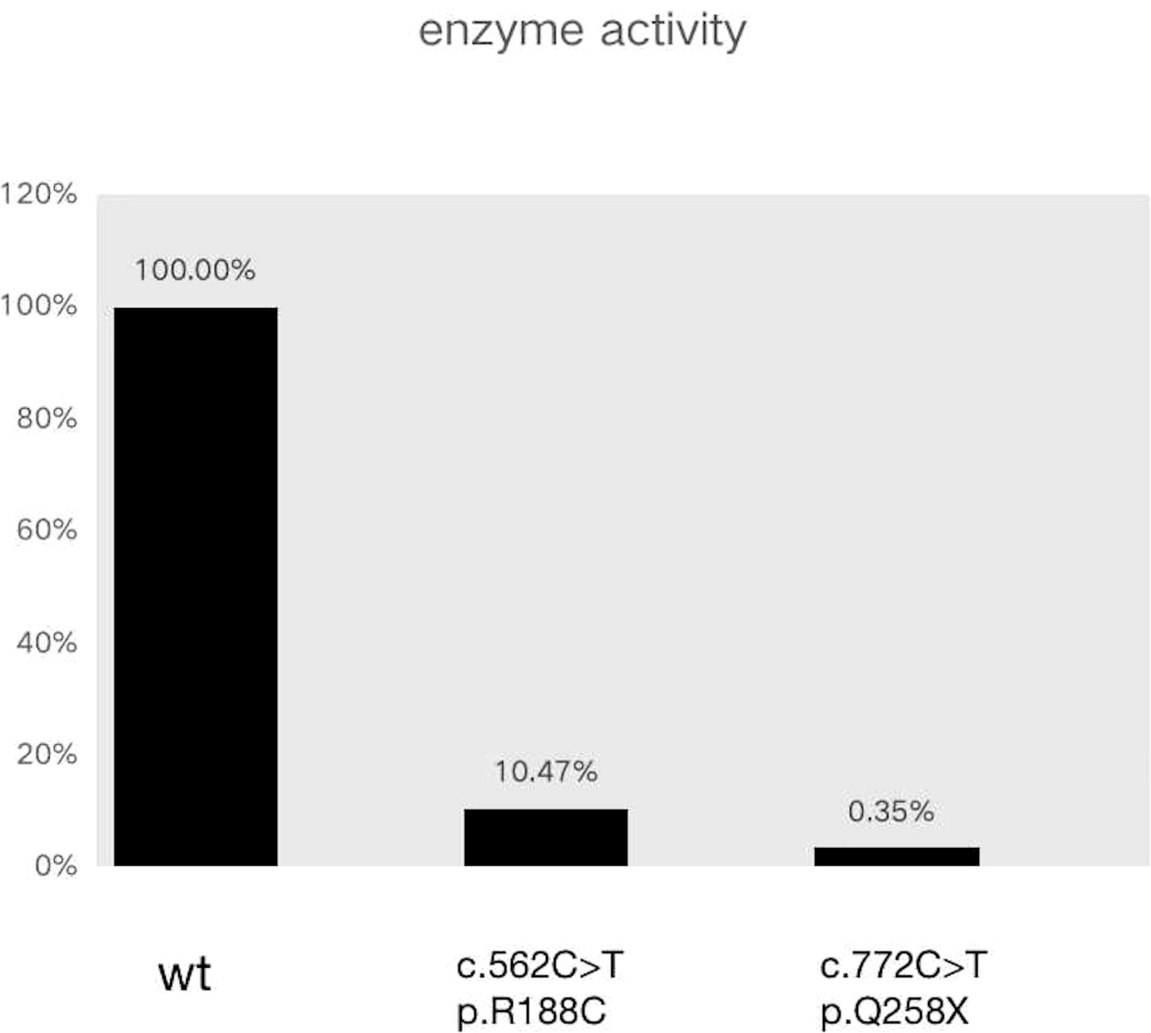
Enzyme activity assay to detect the pregnenolone concentration in this patient.

The results of exome sequencing revealed compound heterozygous *STAR* mutation for c.772C>T (p.Q258X) and c.562C>T (p.R188C). Pedigrees of his family with *STAR* gene mutations and Sanger sequencing confirmation of compound heterozygous mutations analyzed by the direct DNA sequencing are shown in **Figure 1**. The activity of StAR was assessed, and it was found that the wild-type mutation had 0.35%/10.47% activity (**Figure 2**).

### Literature review

**Figure 3:**
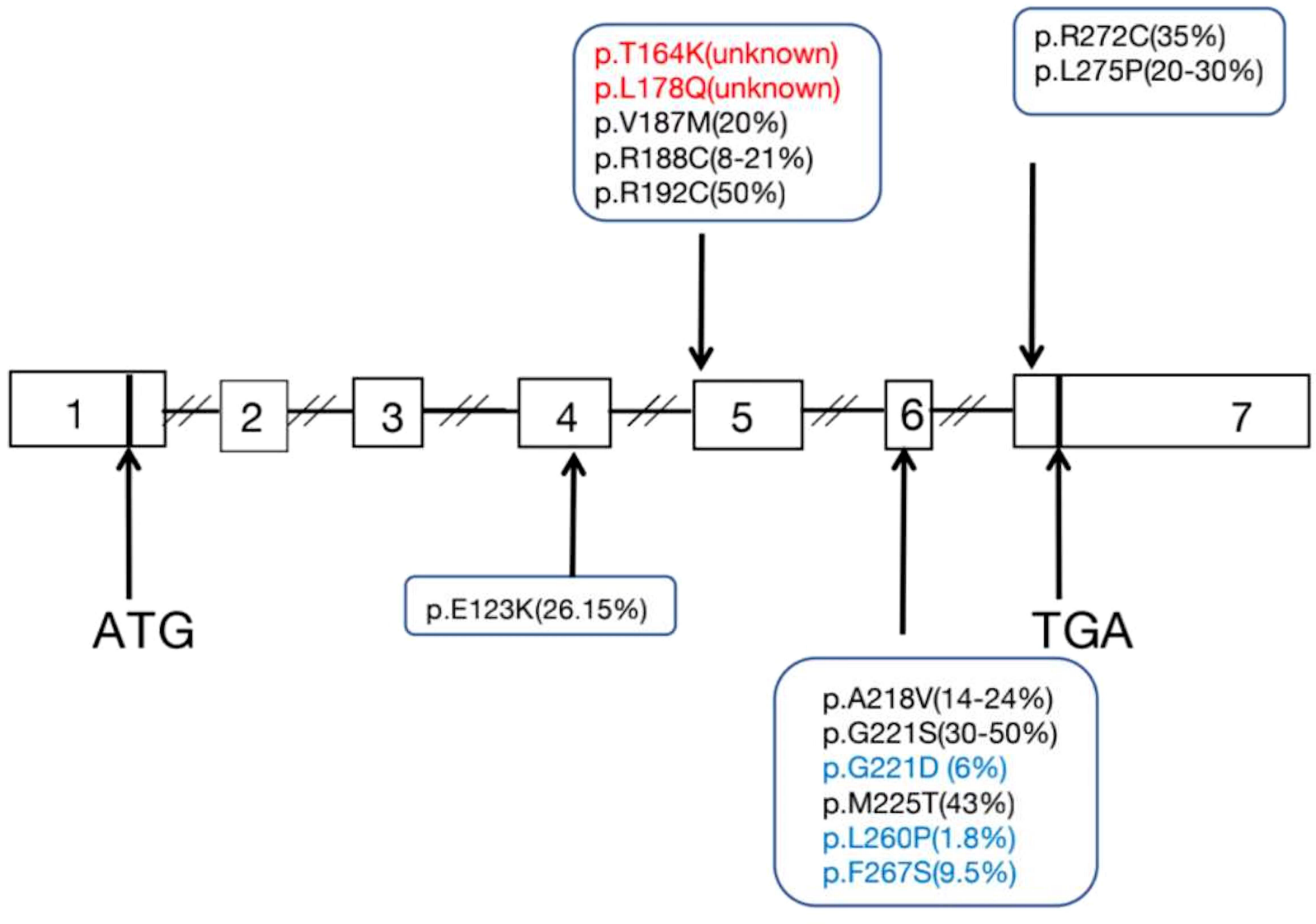
Diagram of the STAR gene showing the location of mutations (Including enzyme activity) identified in patients with non-classic LCAH.

All English and Chinese published articles on non-classic LCAH published until September 2021 and indexed in the PubMed and Wanfang databases were retrieved. In total, 14 mutations in 47 patients from 36 families were found to be associated with non-classic LCAH (**Table 3**) (3–17). As shown in **Table 3**, 46,XY and 46,XX patients accounted for 59.6% (28/47) and 40.4% (19/47), respectively. Moreover, 22 male non-classic LCAH patients with complete male external genitalia (78.5%) and six cases of hypospadias with different degrees (21.5%) were identified. **Figure 3** shows the diagram of the *STAR* gene indicating the location of mutations (including enzymatic activity) identified in patients with non-classic LCAH. These mutations included 14 missense mutations without nonsense, frameshift, and splice-site mutations. Moreover, c.562C>T,STAR-R188C was detected as the most common mutation of *STAR* gene in non-classic LCAH patients, accounting for 37.2%, including 14 homozygous mutations and one compound heterozygous mutation. Enzymatic activity assay showed that c.562C>T,STAR-R188C mutation had reserved 13.6% (3) and 12.8% (4) activity, sufficient to maintain a virilizing phenotype. The other less common mutations included STAR-E123K (2%), STAR-T164K (1%), STAR-L178Q (1%), STAR-V187M (3%), STAR-R192C (7%), STAR-A218V (5%), STAR-G221S (4%), STAR-G221D (2%), STAR-M255T (2%), STAR-L260P (1%), STAR-F267S (1%), STAR-R272C (4%), and STAR-L275P (1%). Enzymatic activity of <10% was found in two patients, including one 46,XY patient with compound heterozygous mutations p.L260P and p.F267S who was born with a micropenis, third-degree hypospadias, severe chordee, a hypoplastic scrotum, and palpable testes, with enzymatic activities of 1.8% and 9.5%; the other case was a 46,XX girl with homozygous G221D mutation who first presented at the age of 11 months with adrenal crisis including vomiting that led to the diagnosis of congenital adrenal hyperplasia (CAH), and an enzymatic activity of 6.3% was recorded.

**Table 3:** Fourteen mutations in 47 patients with non-classic LCAH from 36 families.

		Gender	Nucleotide change	aa change	Age	Residual activity	Reference
F1	46,XX	F	c.559G>A/c.559G>A	p.V187M	4y	21.6%	Baker et al., 2006 (3)
F2-1	46,XY	M	c.562C>T/c.562C>T	p.R188C	2.2y	13.6%	Baker et al., 2006 (3)
F2-2	46,XY	M	c.562C>T/c.562C>T	p.R188C	2.8y	13.6%	Baker et al., 2006 (3)
F3-1	46,XY	M	c.577C>T/c.577C>T	p.R192C	5y	50%	Metherell et al., 2009 (5)
F3-2	46,XX	F	c.577C>T/c.577C>T	p.R192C	?	50%	Metherell et al., 2009 (5)
F3-3	46,XX	F	c.577C>T/c.577C>T	p.R192C	?	50%	Metherell et al., 2009 (5)
F4-1	46,XX	F	c.562C>T/c.562C>T	p.R188C	2y	13.6%	Metherell et al., 2009 (5)
F4-2	46,XY	M	c.562C>T/c.562C>T	p.R188C	58y	13.6%	Metherell et al., 2009 (5)
F5-1	46,XY	M	c.562C>T/c.562C>T	p.R188C	6y	13.6%	Metherell et al., 2009 (5)
F5-2	46,XY	M	c.562C>T/c.562C>T	p.R188C	?	13.6%	Metherell et al., 2009 (5)
F6-1	46,XX	M	c.562C>T/c.562C>T	p.R188C	1.5y	13.6%	Metherell et al., 2009 (5)
F6-2	46,XX	M	c.562C>T/c.562C>T	p.R188C	<1y	13.6%	Metherell et al., 2009 (5)
F6-3	46,XY	F	c.562C>T/c.562C>T	p.R188C	<1y	13.6%	Metherell et al., 2009 (5)
F7	46,XY	glandular hypospadias	c.562C>T/c.562C>T	p.R188C	3y	13.6%	Metherell et al., 2009 (5)
F8	46,XY	hypospadias	c.562C>T/c.562C>T	p.R188C	4y	12.8%	Sahakitrungruang et al., 2010 (6)
F9	46,XY	Ambiguous genitalia	c.6732T>C/c.6753T>C	p.L260P/p.F267S	0	1.8%/9.5%	Sahakitrungruang et al., 2010 (6)
F10	46,XY	Ambiguous genitalia	c.562C>T/c.749C>A	p.R188C/p.W250X	3.5m	12.8%	Sahakitrungruang et al., 2010 (6)
F11	46,XX	F	c.5780G>A/c.5780G>A	p.G221D	12y	6.3%	Sahakitrungruang et al., 2010 (6)
F12-1	46,XX	F	c.661G>A/c.125-126insG	p.G221S/p.T44H_S46X	10m	30-50%	Flück et al., 2011 (7)
F12-2	46,XY	M	c.661G>A/c.125-126insG	p.G221S/p.T44H_S46X	14m	30-50%	Flück et al., 2011 (7)
F13	46,XY	?	/	p.M225T/p.Q258X	10m	43%	Nakae et al., 1997 (8)
F14	46,XY	M	c.950T>C/c.577C>T	p./L275P/p.R192C	2m	24%/50%	Bose et al., 1996 (4)
F15	46,XX	F	c.653C>T/c.950T>C	p.A218V/p.L275P	35d	20%	Bose et al., 1996 (4)
F16	46,XY	M	c.653C>T/c.772C>T	p.A218V/p.Q258X	1m	20%/0	Nakae et al., 1997 (8)
F17-1	46,XY	M	c.653C>T/c.772C>T	p.A218V/p.Q258X	9d	20%/0	Nakae et al., 1997 (8)
F17-2	46,XX	F	c.653C>T/c.772C>T	p.A218V/p.Q258X	35d	20%/0	Nakae et al., 1997 (8)
F18	46,XX	F	c.653C>T/c.772C>T	p.A218V/p.Q258X	13d	20%/0	Nakae et al., 1997 (8)
F19	46,XY	M	c.815G>A/c.772C>T	p.R272C/p.Q258X	5y	35%/0	Ishii et al., 2020 (9)
F20	46,XY	M	c.815G>A/c.772C>T	p.R272C/p.Q258X	4y	35%/0	Ishii et al., 2020 (9)
F21	46,XY	M	c.64_177.del/c.64_177.del	p.G22_L59del	0	0	Ishii et al., 2020 (9)
F22	46,XY	M	c.661G>A/c.653C>T	p.G221S/p.A218V	17m	30-50%/20%	Bae, et al., 2020 (10)
F23-1	46,XX	F	c.553T>A/c.737A>G	p.L178Q/p.D246F	2y	?	Luo et al., 2020 (11)
F23-2	46,XY	M	c.553T>A/c.737A>G	p.L178Q/p.D246F	2y	?	Luo et al., 2020 (11)
F24	46,XX	F	c.562C>T/c.562C>T	p.R188C	14m	12.8%	Burget et al., 2018 (12)
F25	46,XX	F	c.562C>T/c.562C>T	p.R188C	3y	12.8%	Tsai et al., 2016 (13)
F26-1	46,XY	M	c.562C>T/c.562C>T	p.R188C	6y	12.8%	Tsai et al., 2016 (13)
F26-2	46,XY	M	c.562C>T/c.562C>T	p.R188C	9y	12.8%	Tsai et al., 2016 (13)
F27	46,XY	M	c.562C>T/c.562C>T	p.R188C	4y	12.8%	Tsai et al., 2016 (13)
F28	46,XX	F	c.562C>T/c.562C>T	p.R188C	17y	12.8%	Tsai et al., 2016 (13)
F29	46,XX	F	c.815G>A/c.772C>T	p.R272C/p.Q258X	29m	35%/0	Kang et al., 2017 (14)
F30	46,XY	Ambiguous genitalia	Exon1and >50 bp upstream of promoter		0	?	Piya et al., 2017 (15)
F31	46,XY	M	c.815G>A/c.772C>T	p.R272C/p.Q258X	18m	35%/0	Liang et al., 2021 (16)
F32	46,XY	Ambiguous genitalia	c.491C>A/c.707_708delAGinsCTT	p.T164K/p.K236Tfs*47	2.5m	?/<1% (4)	Liang et al., 2021 (16)
F33	46,XY	M	c.306+3_c.306+6delAAGT/661G>A	Intron3-4 fs/p.G221S	13Y	0/17.19%	Zhang et al., 2021 (17)
F34	46,XY	M	c.367G>A/c.465+2T>A	p.E123K/ Intron 4-5	6m	26.15%	Zhang et al., 2021 (17)
F35	46,XX	F	c.367G>A/c.465+2T>A	p.E123K/ Intron 4-5	9m	26.15%	Zhang et al., 2021 (17)
F36	46,XX	F	c.229C>T/c.559G>A	p.Q77X/ p.V187M	2.75y	<1%/20%	Zhang et al., 2021 (17)

d, days; m, months; y, years; F, female; M, male; LCAH, lipoid congenital adrenal hyperplasia.

## Discussion

### 1. Clinical and molecular characterization of the patient

LCAH is a rare and fatal disease caused by loss of functional mutations of StAR. To date, more than 190 patients with LCAH caused by 70 *STAR* mutations have been reported (17). Patients with classic LCAH were typically characterized by lipoid accumulation in the adrenal glands, adrenal insufficiency in infants, and female external genitalia, regardless of karyotype. Some patients with *STAR* mutations developed hyperpigmentation and PAI after infancy, especially 46,XY patients presented with male external genitalia, and they were classified as non-classic LCAH (3). In the present case report, the patient had a micropenis with normal testes and hyperpigmentation at the age of 7 months. Laboratory investigation revealed a high ACTH level and low levels of cortisol and testosterone, and the patient was diagnosed with PAI with signs of mineralocorticoid and glucocorticoid deficiency and adrenal androgen deficiency. Based on the presence of a micropenis and the later onset of hyperpigmentation, along with failed ACTH stimulation test and XY karyotype, we suspected the diagnosis of mutations of *NR0B1*, *CYP11A1*, and *STAR* genes or familial glucocorticoid deficiency (FGD) (*MC2R*, *MRAP*, *NNT*, and *AAAS*). Gene sequencing is the most appropriate diagnostic method to differentiate these diseases. Ishii et al. (9) described non-classic LCAH as either Quigley grade 1 in XY karyotype, no episode of salt losing or requirement of fludrocortisone, or onset of PAI at 1 year or older, while the onset age of PAI is overlapped between non-classic LCAH and classic LCAH (18). In the present study, it was attempted to report a boy with complete male external genitalia who was diagnosed with non-classic LCAH. He had enzymatic activity of greater than 10%, and one girl was diagnosed with classic LCAH with the same mutation as this patient’s, whereas the onset age of PAI was before 1 year old. A previous study demonstrated that the age of onset of non-classic LCAH was after the age of 2 years (2). Therefore, consideration is essential to indicate whether the onset age of PAI is the basic clue for the diagnosis of non-classic LCAH, and the literature review was therefore performed.

### 2. Phenotype and genotype of non-classic lipoid congenital adrenal hyperplasia

Baker et al. (3) in 2006 first reported that LCAH patients with 46,XY karyotype can present with complete male external genitalia, and 14 mutations in 47 patients from 36 families have been identified to cause non-classic LCAH. All previously reported cases were also reviewed to investigate the genotype–phenotype relationship. The mutational analysis showed that c.562C>T(p.R188C) mutation was prevalent in patients with non-classic LCAH, accounting for 37.2% of the total mutant alleles, which could reflect the founder effect on the non-classic LCAH population. It was revealed that the same mutation had different clinical manifestations. We also found that 17 reported patients with c.562C>T homozygous mutation had different phenotypes: among 11 patients with 46,XY karyotype, nine had complete male external genitalia while two had glandular hypospadias, with the onset age of PAI from birth to 48 years; six patients with 46,XX karyotype had different onset ages of PAI (0–17 years). In our previous study (17), patient F34 (46,XY) had complete male external genitalia and with the compound heterozygous variants (c.367G>A/c.465+2T>A,p.Glu123Lys/unknown), in which the enzymatic activity assay revealed that STAR-Glu123Lys was reserved by 26.15%, and the high residual activity could rescue the steroidogenesis capacity of fetal Leydig cells because he had developed male external genitalia although he had first presented at the age of 6 months with adrenal insufficiency after receiving the first dose of vaccine. Patient F35 shared the same mutation as patient F34, while they were from different families, and she had first presented at the age of 9 months with adrenal insufficiency. Patient F36 was diagnosed at the age of 33 months when he presented with pneumonia, and routine investigation showed hyponatremia without hyperkalemia. This case had c.562C>T and c.772C>T mutations, and the residual enzymatic activity could also rescue the steroidogenesis capacity of fetal Leydig cells because he had developed male external genitalia in the presence of a micropenis, and assessment of RAAS revealed a high renin level and a low Ald level without hyponatremia and hyperkalemia; thus, he was diagnosed with non-classic LCAH. Moreover, no significant correlation between genotype and phenotype of non-classic LCAH was identified.

### 3. 46,XY male patients with non-classic lipoid congenital adrenal hyperplasia

It is widely accepted that the current classification criteria for non-classic LCAH include late age of onset (generally defined as onset after age of 2–4 years) and external genital development that is consistent with the chromosomal karyotype (2). The majority of LCAH patients presented with female external genitalia, while few cases were reported with male external genitalia that were diagnosed as non-classic LCAH. A high residual activity could rescue the steroidogenesis capacity of fetal Leydig cells because these patients develop male external genitalia. The clinical phenotype is related to the residual enzymatic activity, in which the enzymatic activity >10%–20% is considered as non-classic LCAH and the enzymatic activity <10% is considered as classic LCAH (9). The literature review resulted in the identification of 28 46,XY patients, of whom 22 cases had complete male external genitalia (78.5%), their residual enzymatic activity was not more than 20%, even was <10%, and the onset age of PAI was 0–58 years; there were six cases with different degrees of hypospadias (21.5%), in which their residual enzymatic activity was ≥10% and even >20%. Therefore, a higher residual enzymatic activity can maintain the formation of male external genitalia, while it does not necessarily indicate that the onset age of PAI is late, and it could be affected by several factors, such as stress, temperature, sodium intake, and StAR-independent steroid pathways. It is noteworthy that 46,XY patients can be diagnosed before the age of 1 year, and the onset age of the same mutation is also different in classic and non-classic cases. Thus, the onset age can be used as one of the diagnostic indicators of non-classic LCAH, but not the only one.

The clinical phenotypes of non-classic LCAH are highly variable. Routine physical examination, laboratory measurement, genetic testing, and, importantly, enzymatic activity assay may facilitate the early diagnosis of non-classic LCAH. Further study is required to verify the findings of the present case report.

## Data availability statement

The original contributions presented in the study are included in the article/Supplementary Material. Further inquiries can be directed to the corresponding authors.

## Ethics statement

The studies involving human participants were reviewed and approved by Ruijin Hospital Ethics Committee, Shanghai JiaoTong University School of Medicine. Written informed consent to participate in this study was provided by the participants’ legal guardian/next of kin.

## Author contributions

WL, TZ, and LZ as co-first authors wrote and translated this article. XW, JW, LY, YX, ZD, WW, and SS collected all the clinical information of these patients. RH, GN, CL, and XM are the corresponding authors who edited and reviewed this manuscript and approved the version to be published.

## Conflict of interest

The authors declare that the research was conducted in the absence of any commercial or financial relationships that could be construed as a potential conflict of interest.

## Publisher’s note

All claims expressed in this article are solely those of the authors and do not necessarily represent those of their affiliated organizations, or those of the publisher, the editors and the reviewers. Any product that may be evaluated in this article, or claim that may be made by its manufacturer, is not guaranteed or endorsed by the publisher.
